# High Temperature Fracture Resistance of Model Kraft Recovery Boiler Deposits

**DOI:** 10.3390/ma15144759

**Published:** 2022-07-07

**Authors:** Mohammad Abdully, Nikolai DeMartini, Markus Bussmann, Mark Kortschot

**Affiliations:** 1 Department of Mechanical and Industrial Engineering, University of Toronto, 5 King’s College Rd., Toronto, ON M5S 3G8, Canada; mohammad.abdully@mail.utoronto.ca (M.A.); markus.bussmann@utoronto.ca (M.B.); 2 Department of Chemical Engineering and Applied Chemistry, University of Toronto, 200 College St., Toronto, ON M5S 3E5, Canada; nikolai.demartini@utoronto.ca

**Keywords:** recovery boiler, deposit, fouling, fracture, sintering, sootblower, heat exchanger

## Abstract

In kraft paper mills, supersonic steam jets are used to remove deposits that build up on the heat exchanger tubes in the recovery boiler. In this study, the fracture toughness *K_C_* and work of fracture, *W_F_*, of simulated boiler deposits were measured at temperatures up to 500 °C to determine the optimal conditions for deposit removal. The model deposits experienced an important brittle to ductile transition at ~450 °C. Above this temperature, ductile deposits required lower peak force, but four times more energy to fracture when compared to those tested at lower temperatures. The transition was clear in scanning electron micrographs of the fracture surfaces. The findings have significant implications for mills wishing to optimize sootblower performance.

## 1. Introduction

The pulp and paper industry produces about 400 million tons of paper and paperboard worldwide every year [1]. Fine paper is made using the Kraft process, in which wood chips are delignified using an aqueous solution of sodium hydroxide (NaOH) and sodium sulphide (Na_2_S) in a pressurized digester [2]. This process produces cellulose-rich pulp and weak black liquor (alkaline solution of dissolved organics and pulping chemicals) as a byproduct. In order to recover the chemical energy in this liquor and the inorganic pulping chemicals, the black liquor is first concentrated in an evaporator and then burned in a recovery boiler. The inorganics flow out of the bottom of the recovery boiler as molten smelt and are dissolved in water, yielding green liquor, which is processed further to regenerate the liquor used in pulping. The black liquor burned in the recovery boiler is a valuable resource that generates 250 MW to 500 MW for a typical kraft mill [1], providing a significant fraction of the electricity needs of the mill.

To recover the heat of combustion, hot flue gases pass through heat exchangers to generate and superheat steam. Fouling occurs in the tube banks due to deposition of black liquor droplets carried up with the combustion gases, and from the condensation of salt vapours (fume) [3]. These deposits insulate the heat exchanger tubes, reducing heat transfer efficiency, and eventually, the tube banks can become completely plugged, requiring a full plant shutdown.

In order to mitigate the deposit accumulation, a high-pressure steam jet is used to blast the surface of boiler tubes to erode, fracture, and dislodge the deposits [4]. The device delivering this jet is known as a sootblower, and consists of a long steel tube, or lance, with a specialized set of nozzles that directs a pair of opposing jets at 90° to the long axis of the lance. The lance rotates while traversing between banks of heat exchanger tubes, and the jets remove some or all of the deposits during this process.

A typical industrial kraft recovery boiler will have up to 100 sootblowers to reduce fouling in multiple locations. This process is very energy intensive, using 3 to 12% of the total steam produced by the boiler [5], so even a small improvement in sootblower efficiency would result in immense economic savings. As a result, many studies of deposit formation and removal have been performed. For example, modern sootblowers have nozzles specially designed to produce a fully expanded supersonic jet in order to eliminate the energy lost to a shockwave with a less sophisticated nozzle [6].

There has been quite a bit of work on the causes of deposit formation. However, in spite of the economic significance of efficient deposit removal, there have been few studies of the actual mechanics of deposit erosion, fracture, and debonding, and no measurements of the corresponding material properties at realistic boiler temperatures. In this study, experiments to determine critical material properties at temperatures up to 500 °C were conducted. By understanding the properties that govern fracture at high temperatures, we can provide guidance for optimizing sootblower operation.

## 2. Background

During sootblowing, deposit removal can involve gradual erosion or sudden brittle fracture and debonding of large chunks of deposit [7]. Deposits can also debond if there is significant tube bending or vibration or if a thermal shock is induced by differential cooling. These mechanisms are controlled by a balance between the applied forces (from the jet, differential cooling, etc.) and the corresponding material properties. The material properties are principally governed by the chemistry of the black liquor and the temperature and flow conditions in the boiler.

**Figure 1:**
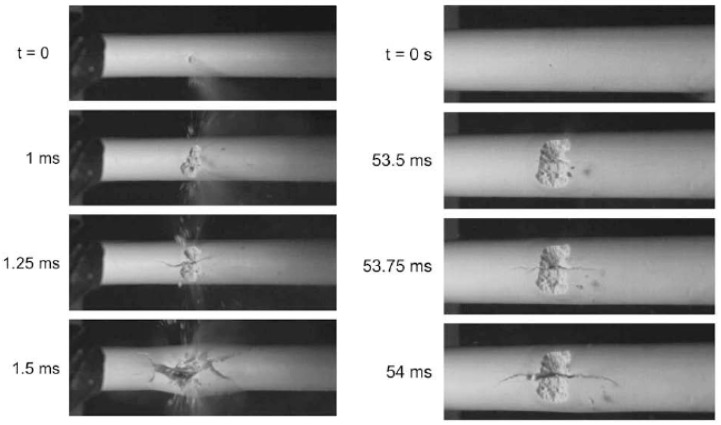
Breakup images for a thin deposit (**left**) and thick deposit (**right**). (Reprinted from [8] with permission from Elsevier).

Previous studies have addressed both sides of the failure equation: the applied loading and the material properties. The breakup/debonding model of deposit removal during jet impingement was studied by Pophali et al., who conducted a model experiment using a supersonic jet impacting a ¼ scale model deposit made of gypsum while imaging the breakup at high speed as depicted in Figure 1 [8]. The authors used two deposit configurations, thin and thick, to investigate the fracture pattern induced by jet impingement. The fracture pattern for both deposits followed the same sequence: erosion leads to a pit, which is subsequently pressurized by the jet, causing circumferential tensile force, axial cracks, and finally debonding [9]. The time required for this sequence was much longer for thicker deposits, but the sequence itself was unchanged. Although the fast fracture nature of this process implies that a conventional fracture mechanics failure criterion should be used to predict failure, this has not previously been demonstrated in the literature.

The deposits can also be stressed to failure by creating temperature differentials, a process referred to in the industry as thermal shedding. This is done by temporarily decreasing boiler temperature by reducing the black liquor flow. The change in gas temperature results in small microcracks and differential cooling between the tube and deposit, causing deposits to debond and fall off [10]. The thermal shedding mechanism is most effective in the superheater region, where most deposits are primarily removed during the first two hours [10]. Ozcan et al. simulated thermal shedding by inducing controlled thermal stresses in a deposit specimen to investigate the decrease in deposit strength [11]. Deposits that were thermally shocked using various quench cycles were as much as 50% weaker than control specimens due to the introduction of microcracks. The fact that thermal shocking and the resultant microcracks can be so significant strongly suggests that a fracture mechanics approach should be used to predict failure.

The material properties which would determine whether a deposit fails at a particular stress state depend on both boiler conditions and the chemical composition of the deposits [12]. For example, Mao et al. used a synthetic mixture of chloride, potassium, and carbonate to create a deposit on a probe inside a 400 °C furnace. It was found that increasing concentrations of potassium and carbonate created deposits that were difficult to remove. Many other studies of the relationship between deposit chemistry and properties have been carried out (e.g., [13]).

Deposits can form by direct deposition and sticking of discrete “carryover” particles entrained in the exhaust gases, or through condensation of volatile compounds on the relatively cool heat exchanger tubes. In both cases, deposited particles fuse through sintering to form the problematic solid deposits that plug the heat exchanger tube banks. Tran et al. [14] studied the sintering temperatures of condensates formed under typical boiler conditions and found that sintering began at temperatures of 300 °C. The authors also found that at temperatures within the range of 500 °C to 600 °C, the deposits sintered rapidly, reaching maximum strength in 1 h [14].

To study the sintering of carryover deposits, Tran et al. [14] used synthetic mixtures that were melted, cooled, powdered, and pressed into bars of 3 by 5 mm cross-sections and 50 mm in length. The bars were sintered in a furnace at various temperatures for one hour, and the change in the bending strength with sintering temperature was measured. The bars were loaded in a three-point bend test at room temperature, a test that measures the unnotched tensile strength of the material. The maximum bending strength was found in samples with 20% NaCl content sintered at 600 °C as 10 MN/m^2^.

## 3. Predicting Failure

Brittle materials, such as chalk, glass, and many ceramics, fail by fast fracture, where a crack propagates through the material at something approaching the speed of sound. For these materials, the maximum stress is not constant at failure and therefore cannot be used to predict failure. Instead, a particular combination of stress and crack length has been experimentally found to be constant at the moment of failure, regardless of the specimen geometry. The stress intensity factor, *K*, has been found to be sufficient to characterize the entire stress field around a sharp crack, and when it reaches a critical value, known as the fracture toughness of the material, *K_C_*, the crack will invariably propagate and the material will fail. The stress intensity factor is expressed as

(1) $$K=Y\sigma_{\infty }\sqrt{\pi a}$$

where $\sigma_{\infty }$ is the uniform tensile stress far from a crack, *a* is the crack length, and *Y* is a factor accounting for the overall specimen geometry (*Y* = 1 for a central crack of length 2a in an infinitely large planar specimen).

At the moment of failure, *K = K_c_*, so Equation (1) can be rearranged to solve for the remote stress at failure, $\sigma_{F}$. For materials failing by fast fracture and subject to certain constraints, the observation that *K = K_c_* at failure is an experimental observation. The reason that this should be so relates to a basic energy balance proposed by A.A. Griffiths in 1921, and the interested reader is referred to [15] for more details.

(2) $$\sigma_{F}=\frac{K_{c}}{Y\sqrt{\pi a}}$$

To measure the fracture toughness of a material, a cracked specimen is loaded to failure, and the *K* at failure is computed using Equation (1). For example, this can be done in three-point bending, where the effective *Y* can be computed for a particular beam and crack geometry analytically.

**Figure 2:**
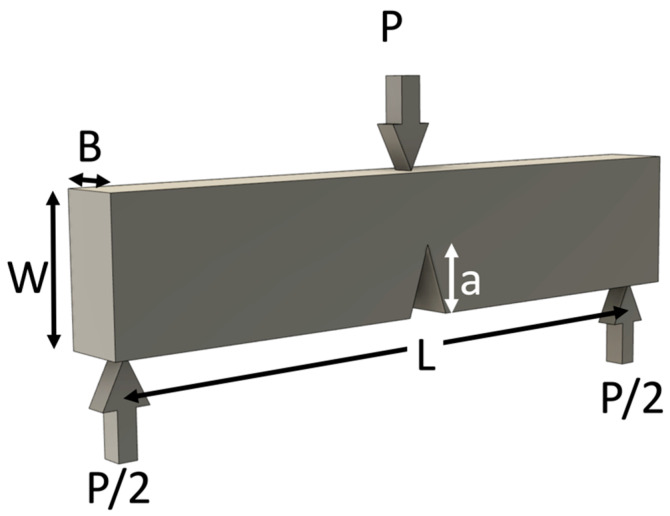
Test to measure fracture toughness using the bend test with a pre-notched specimen.

In order to predict the failure of a cracked brittle material subjected to load, it is necessary to compare the stress intensity (for that particular geometry and load) to the fracture toughness measured using a standardized geometry such as the one shown in Figure 2.

A recovery boiler operates at high temperatures, resulting in deposits that vary in mechanical properties depending on the section of the boiler in which they form. Deposits in the superheater are exposed to temperatures significantly higher than those in other sections. The fracture toughness can vary with temperature [16], but at higher temperatures, deposits behave in a ductile fashion and *K* is no longer expected to be constant at failure [17]. When a material displays significant ductility, more sophisticated elastic/plastic fracture mechanics are needed to predict failure, although these approaches are beyond the scope of this work. In this study, we measured the work of fracture to provide some insight into fracture behaviour for softer materials displaying significant ductility.

## 4. Experimental Methods

It is not possible to obtain actual recovery boiler deposit samples from a commercial mill without introducing microcracks during the cooldown needed to remove and transport them. Thus, in this study, sintered precipitator ash was used to create model recovery boiler deposit materials. Two commercial kraft mills (Mill A and Mill B) provided samples of precipitator ash, as presented in Table 1. Differential scanning calorimetry (DSC) (STD650 Simultaneous Thermal Analyzer, TA Instruments, New Castle, DE, USA) was used to measure the melting temperatures. Ash composition was also characterized using ion chromatography with conductivity detection (Dionex Integrion HPIC, ThermoFisher Scientific, Waltham, MA, USA) to analyze Na, K, SO_4_, and Cl. For cations, the Dionex IonPac CS19, 4 × 250 mm column and Dionex EGC 500 MAS (Methanesulfonic acid) eluent were used. For the anions, the Dionex IonPac AS18, 2 × 250 mm column, and Dionex EGC 500 KOH (potassium hydroxide) eluent were used. Carbonate was analyzed by a total inorganic carbon analyzer (Shimadzu TOC-V_CPN_, Columbia, MD, USA).

**Figure 3:**
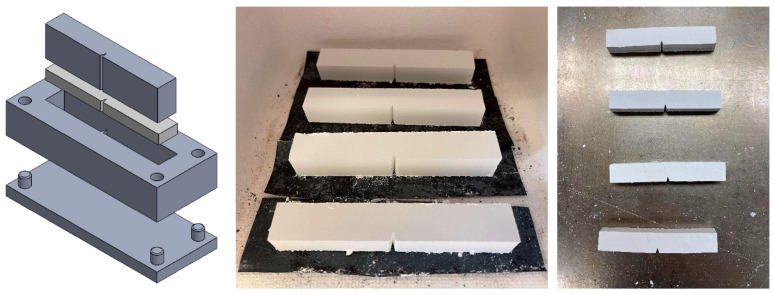
Powdered ash compressed into mold (**left**), compressed specimens placed in the furnace (**middle**), sintered deposit specimens removed from the furnace (**right**).

In order to create specimens for fracture testing, precipitator ash was introduced into the cavity of a three-part compression mold, shown in Figure 3. The mold consists of a base, frame, and punch. First, the base and frame were assembled and loaded with 40–45 g of precipitator ash. The punch was then inserted to compact the ash using ~500 N of manual compressive force. After compaction, the base was removed, and the compacted specimens had enough structural integrity to be punched through to a flat stainless-steel sheet, which was then transferred to a sintering furnace.

**Figure 4:**
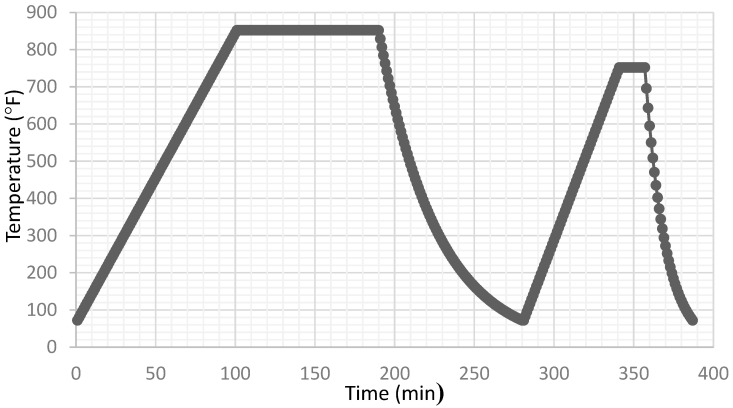
Sintering time–temperature profile for creating and testing deposit specimens.

The sintering time/temperature cycle was relatively long in order to minimize thermal gradients in the specimens. Rapid heating or cooling was found to result in immediate fracture. Two different time–temperature profiles were used to achieve different levels of sintering, Table 2. The three-point bend environmental chamber and the sintering oven were one and the same and specimens remained in the oven between sintering and testing, with the door open only long enough to transfer one specimen from the sintering tray to the three-point bend apparatus. In Figure 4, the first plateau represents sintering and the second represents the testing temperature. The specimens were quite brittle in the sintered state, so they were handled carefully during the transfer to the bending apparatus. Figure 3 shows that the specimens were not perfectly flat after sintering, but this would not have an influence on the measured failure loads or fracture toughness.

**Figure 5:**
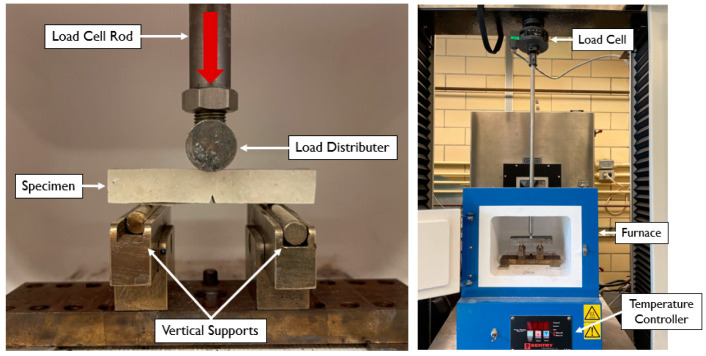
Experimental three-point bend test apparatus.

The sintered specimens were tested at room temperature and then various temperatures between 400 °C and 500 °C. Figure 5 shows the experimental three-point bend platform, which sits in the oven during testing. Load was introduced through a long rod which runs through a hole in the top of the oven to the load cell. A cylindrical load distributor was threaded at the end of the load cell rod and spanned the entire width of the specimen. A 100 kN Sintech tensile test machine was used to introduce the compressive loads.

Specimens were measured carefully after testing to obtain the dimensions needed to calculate the critical stress intensity factor (*K_C_*) using Equation (3), which is a specific instance of Equation (2) computed analytically for a three-point bend specimen. The work of fracture (*W_F_*) was also computed as the total area under the force displacement curve divided by the cross-sectional area of the specimen minus that of the notch.

(3) $$K_{C}=\frac{4P}{B}\sqrt{\frac{\pi }{W}}\left[1.6{\left(\frac{a}{W}\right)}^{\frac{1}{2}}-2.6{\left(\frac{a}{W}\right)}^{\frac{3}{2}}+12.3{\left(\frac{a}{W}\right)}^{\frac{5}{2}}-21.2{\left(\frac{a}{W}\right)}^{\frac{7}{2}}+21.8{\left(\frac{a}{W}\right)}^{\frac{9}{2}}\;\right]$$

where the symbols were defined in Figure 2.

The number of specimens tested for each sintering condition and test temperature varied. Details are provided in Appendix A.

## 5. Results and Discussion

Sintering resulted in shrinkage, densification, and the development of strength and stiffness. The density of the unsintered compressed ash sample was difficult to control and ranged from 0.800 g/cm^3^ to 1.00 g/cm^3^. The sintered density was measured using dimensions taken after the fracture testing was completed and ranged from 1.80 g/cm^3^ to 2.20 g/cm^3^. These densities are within the range of densities that Tran et al. reported for sintered ash samples [14]. Samples tested at temperatures above 450 °C did undergo some additional sintering as the oven heated up beyond the sintering onset temperature.

The sintered ash specimens were tested at various temperatures to develop the fracture toughness versus temperature profile. The main range investigated was 400 °C to 500 °C, because these temperatures correspond to those found in the regions with extensive fouling: at the back end of the superheater bank and inlet of the boiler bank. It is important to note that the sintering parameters were kept constant during these tests so that only the effect of testing temperature on *K_C_* was measured.

**Figure 6:**
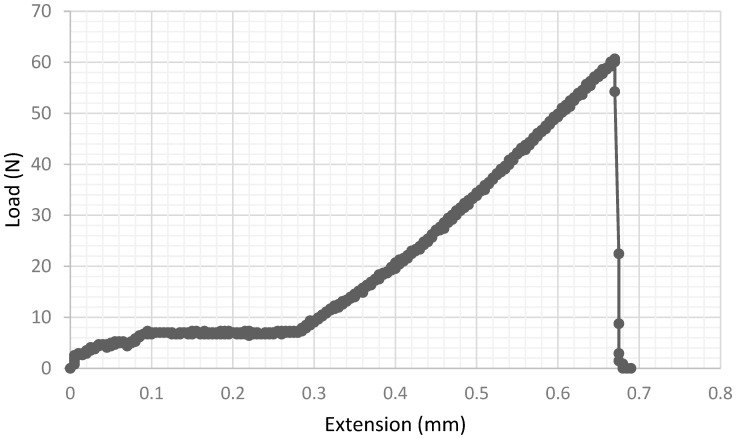
Force—extension curve for a brittle deposit tested at 400 °C.

Figure 6 shows the force versus extension curve for a typical specimen that broke by brittle fast fracture. After an initial plateau, which can be attributed to settling of the loading noses to match the imperfect surfaces of the sintered specimens, the curve is essentially linear to failure.

**Figure 7:**
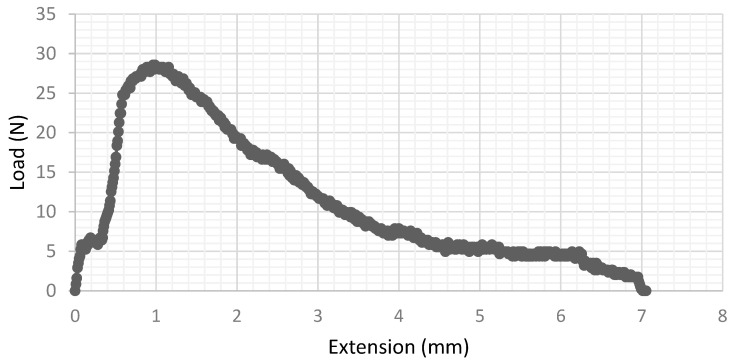
Force–extension curve for a ductile deposit tested at 460 °C.

When the testing temperature was increased past 450 °C, the specimens no longer fractured suddenly, but instead experienced a slow and ductile tearing crack extension. A typical result is depicted in Figure 7, where the total deformation is ~7 mm and the load decline corresponding to crack extension starts at ~1 mm of deformation.

**Figure 8:**
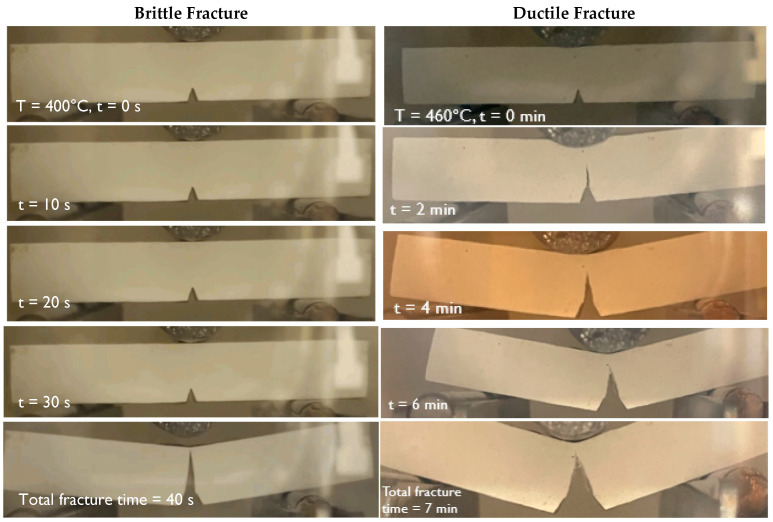
Comparison of two specimens tested at 400 °C and 460 °C, respectively.

Time lapse images of the two types of fracture are shown in Figure 8. For the brittle specimen tested at 400 °C, there was no crack extension until the instant of complete fracture. For the ductile specimen, tested at 460 °C, the crack growth was stable. Both of these tests were run at a crosshead speed of 1 mm/min, so the time in minutes corresponds to a displacement in mm.

**Figure 9:**
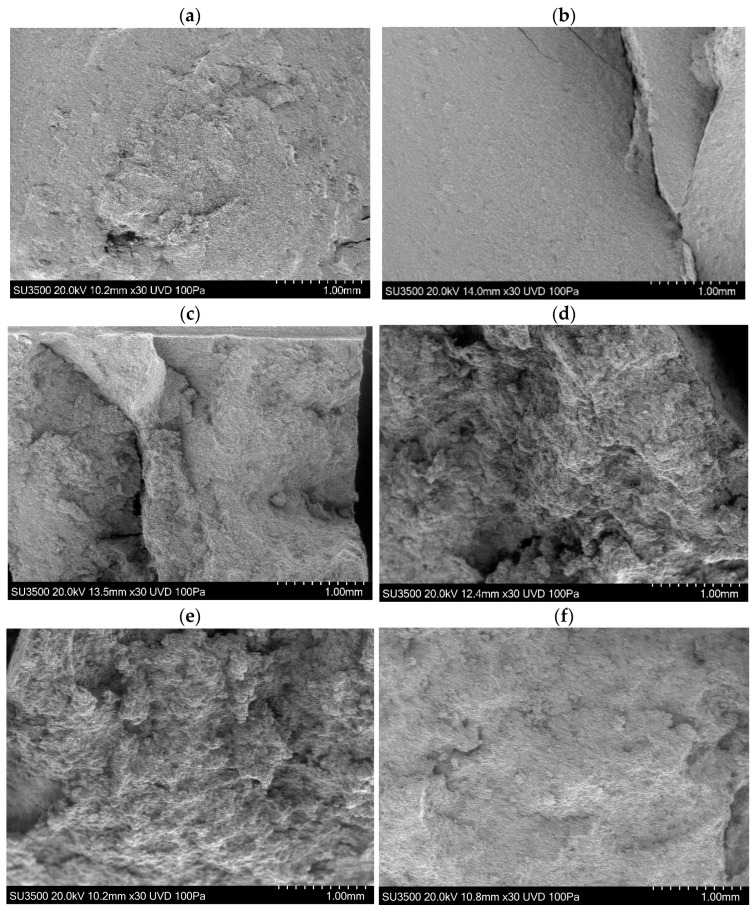
SEM Images of Ash A displaying the brittle–ductile transition. (**a**) Specimen tested at 400 °C—Brittle; (**b**) Specimen tested at 450 °C—Brittle; (**c**) Specimen tested at 450 °C—B/D; (**d**) Specimen tested at 450 °C—Ductile; (**e**) Specimen tested at 475 °C—Ductile; (**f**) Specimen tested at 500 °C—Ductile.

Figure 9 shows fracture surfaces for specimens from Mill A, fractured at temperatures from 400 °C to 500 °C. There is a clear change in the fracture surfaces as the temperature increases, consistent with a transition from brittle to ductile behaviour observed in the force displacement curves. The specimens tested at 400 °C had very smooth, faceted fracture surfaces consistent with a fast fracture and little plastic deformation. Note that it is plastic deformation on either side of the crack plane that consumes energy during fracture, leading to higher fracture toughness and work of fracture. The absence of any deformation on the fracture surface is therefore expected to correspond to low toughness specimens, and this was true in our study. Specimens tested at 475 °C had comparatively rough fracture surfaces, with clear evidence of ductile tearing and plastic flow. This deformation consumes energy, leading to higher toughness and work of fracture. Specimens of ash from Mill A tested at 450 °C could fail by either fast fracture or ductile tearing, and hence 450 °C is regarded as the brittle/ductile transition temperature. Finally, specimens tested at 500 °C showed signs of ductile tearing; however, there is a marked transition back to a smoother surface with less plastic deformation. At 500 °C, these specimens were partially melted, reducing the work of fracture and tensile strength of the material. Even though significant plastic deformation accompanies the fracture plane advance at 500 °C, this deformation does not take as much energy at this high temperature.

**Figure 10:**
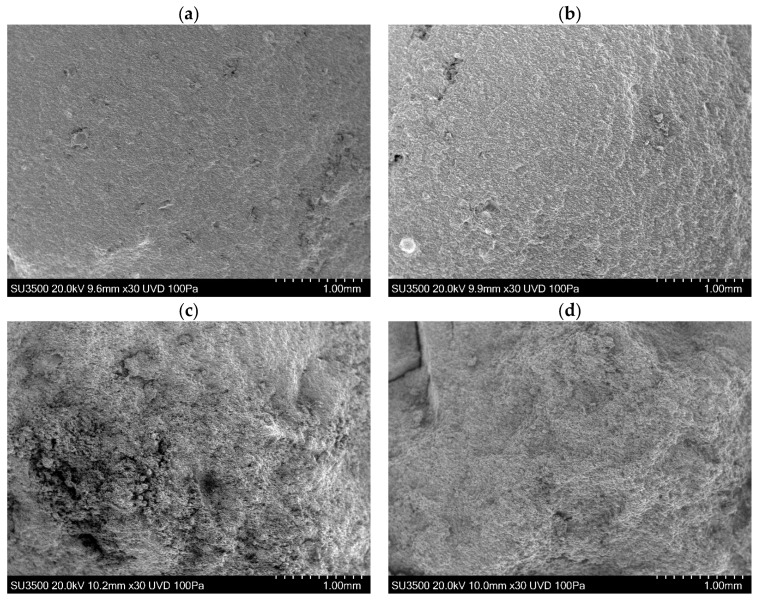
SEM Images of Ash B with different chemical composition. (**a**) Specimen tested at 400 °C—Brittle; (**b**) Specimen tested at 460 °C—Brittle; (**c**) Specimen tested at 475 °C—Ductile; (**d**) Specimen tested at 500 °C—Ductile.

A comparable set of Images for the specimens from Mill B are shown in Figure 10. They are similar, although it appears that the brittle transition is shifted to a slightly higher temperature, presumably due to their slightly different chemical composition.

The peak load for all three-point bend tests was used to calculate the stress intensity factor at the onset of crack growth, using Equation (3).

**Figure 11:**
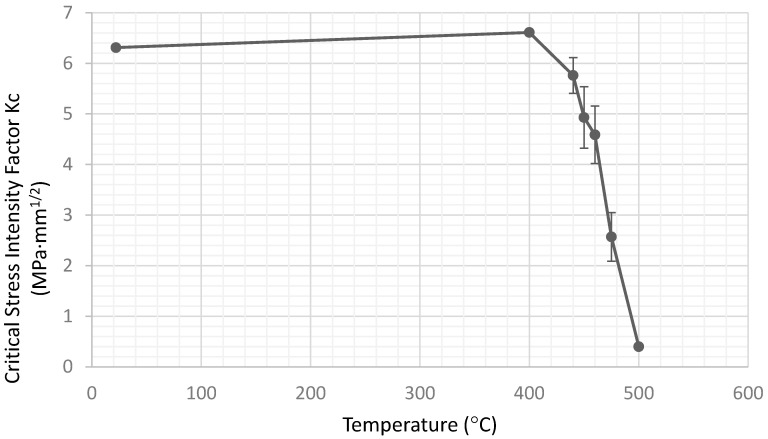
Critical stress intensity factor versus testing temperature.

The critical stress intensity factor, or fracture toughness, is plotted as a function of testing temperature in Figure 11. There is no significant difference between specimens tested at 400 °C and those tested at room temperature, but the computed *Kc* drops above 400 °C as the measured peak load at crack extension drops. The results suggest that some phases of the sintered precipitator ash are softening or melting beginning at about 400 °C. The material is solid, but substantially weaker at 500 °C, and beyond this temperature, it would begin to sag under its own weight and could not be tested.

For specimens above 450 °C, which do not fail by fast fracture, it is more appropriate to compute a work of fracture, or energy under the stress–strain curve. In fact, the computed *Kc*, while proportional to the relative peak load reached, cannot be used to predict fracture for ductile materials, and strictly speaking, should not be plotted in Figure 11. [15,18] The corresponding work of fracture data representation of the data will be presented shortly.

Both the sintering conditions and chemical composition were varied slightly to determine how stable or representative the *Kc* values were. Table 2 presented the original sintering conditions used to plot the results in Figure 11 and a second or “extended” sintering condition.

**Figure 12:**
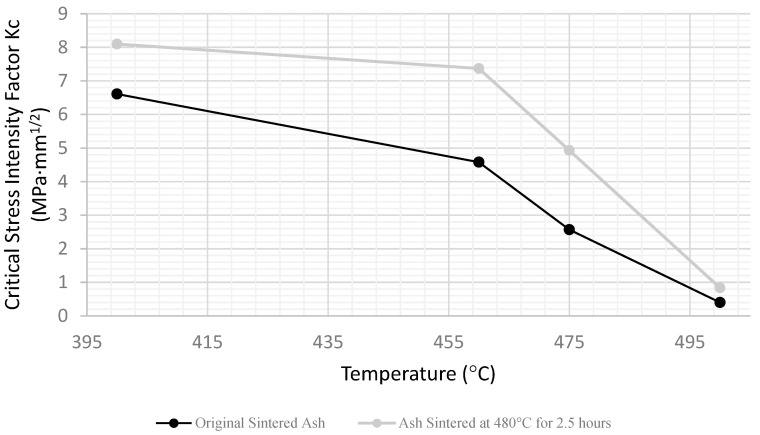
Critical stress intensity factor versus temperature for original sintered ash and extended sintered ash.

Figure 12 compares the results for the two sintering conditions, and as expected, the specimens sintered at a higher temperature and for longer were tougher throughout the range; however, the general shapes of the curves are similar. The sintered precipitator ash specimens are only models for real boiler deposits, so the trends are the focus.

**Figure 13:**
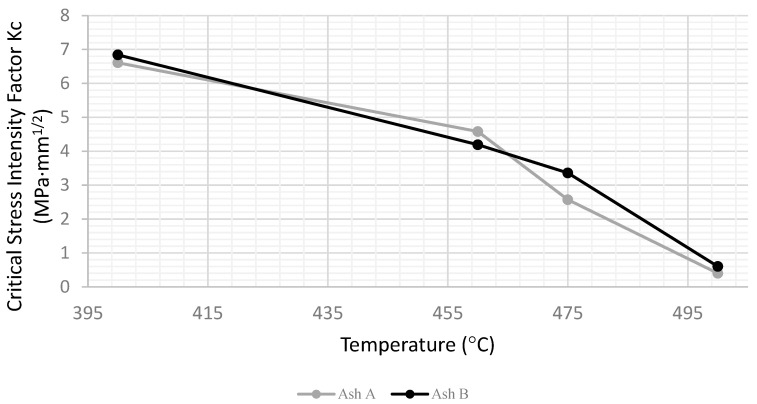
Critical stress intensity factor versus temperature of Ash A and Ash B.

Figure 13 illustrates the effect of varying ash composition on the critical stress intensity factor using ashes from the two mills. There is very little difference in performance, perhaps because the chemical compositions were not very different from each other.

The area under the force–extension curve has dimensions of energy, and for a ductile fracture, this number is divided by the new crack area created and referred to as the work of fracture *W_F_*. Rather than measuring peak force, *W_F_* is a measure of the total energy that must be externally supplied to drive a crack in ductile materials. A short blast of steam from a sootblower might provide a momentary peak impact pressure that results in some fracture, but in the ductile range, at temperatures over 450 °C (for these materials), the duration of the application of load and total steam energy delivered are also likely to be important.

**Figure 14:**
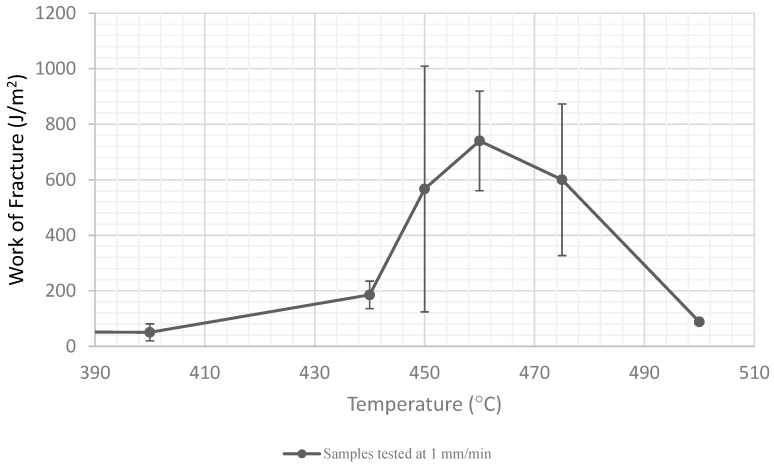
Work of fracture versus testing temperature.

The area under the stress–strain curves at all temperatures is normalized by the new crack area and plotted as *W_F_* in Figure 14. At lower T, the failure is brittle and *W_F_* is low because the displacement is very small (<1 mm in our tests). As the material is heated, it begins to show some ductility, which, although reducing the peak force at crack initiation, increases the work of fracture. At these intermediate temperatures, crack propagation is a ductile tearing process, and significant energy is consumed in plastic zones on either side of the propagating crack tip. *W_F_* drops again at very high T as the material approaches its melting point and the crack propagation does not result in significant deformation of the material surrounding the crack. At ~450 °C, the deposit is transitioning from a brittle to a ductile failure, and some specimens failed in each mode, resulting in a very high standard deviation. Note that the peak load was about the same, so the *Kc* plot had the same standard deviation at 450 °C as at other temperatures.

These trends in work of fracture are clearly visible in the fracture surfaces presented in Figure 9 and Figure 10. In Figure 9d,e for example, there is clear plastic deformation of the material on either side of the running crack. This deformation costs energy, which is reflected in the *W_F_* curve.

**Figure 15:**
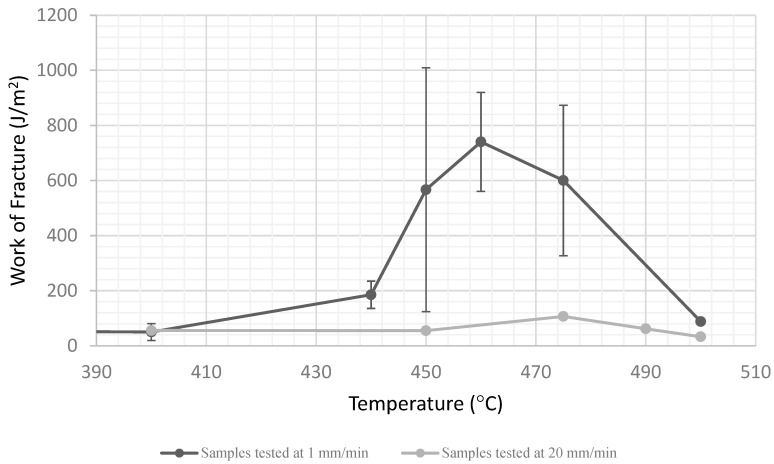
Critical strain energy release rate versus temperature for specimens tested at two different crosshead speeds.

The data reported thus far were collected during tests performed with a crosshead speed of 1 mm/minute. Sootblowers deliver high-speed pressure pulses, and strain rate is known to significantly affect the response of viscoelastic materials. High strain rates tend to suppress the time-dependent plastic flow processes and make materials behave in a less ductile, more brittle fashion. In order to determine if this effect was present in the sintered ash specimens, an additional set of tests were run at 20 mm/min, the maximum speed available on our testing machine. The data are presented in Figure 15. As expected, the total amount of plastic flow and energy absorption was reduced, and the peak in the energy absorption was shifted to higher temperatures. Both of these effects are expected for a viscoelastic material. Although the peak is lower for the specimens tested at higher speeds, it should be noted that at least double the energy is needed to break the specimens at the peak than at a lower temperature.

## 6. Conclusions and Industrial Implications

In critical sections of the recovery boiler, the back end of the superheater and front of the generating bank, the temperatures are such that tough boiler deposits form and are difficult to remove by sootblowing. The data presented here show that in the range of 450 °C to 500 °C, model deposits had a reduction in peak stress at failure but went through a maximum in their work of fracture, or capacity to absorb energy during cracking. At low temperatures (below 450 °C), the model deposits were relatively brittle, and this implies that fast fracture could be induced by a short, high-pressure blast from a sootblower jet. At high temperatures (above 480° C), the peak impact pressure could be much lower, but more time would be required to dislodge a deposit, as it becomes much more ductile nearer the melting temperature.

The most interesting finding is that there is an intermediate range where the fracture toughness is still high and there is a maximum in the work of fracture. This implies that a long duration, high-pressure jet would be needed to initiate and propagate the fracture needed to remove a deposit in this range. It is no surprise that this is the temperature range corresponding to the boiler locations where fouling is most problematic. Of course, experiments in this study were conducted with sintered precipitator ash, and both the magnitude and temperature range for this effect would be expected to vary somewhat for real deposit material and from mill to mill.

One deposit removal strategy that is sometimes used by mills is to temporarily restrict black liquor flow in order to drop the boiler temperature and introduce a thermal shock. (Gas temperatures may drop by several hundred degrees in the superheater region.) The data from this study suggest that a drop of 80–100 °C, combined with intense sootblower activity in the critical boiler regions, might be successful in dislodging deposit material. This would be an interesting experiment to conduct in a mill environment.

## Figures and Tables

**Table 1:** Chemical analysis of Ash A and Ash B.

Chemical Composition	Ash A	Ash B
Sodium, Na (eq/kg ash)	12.3	12.0
Potassium, K (eq/kg ash)	1.5	2.0
Sulphate, SO_4_ (eq/kg ash)	9.6	11.4
Carbonate, CO_3_ (eq/kg ash)	4.3	2.4
Chloride, Cl (eq/kg ash)	0.3	0.4
Cation/Anion	0.977	0.986
First Melting Temperature, T_o_	512.7 °C	513.6 °C

**Table 2:** Sintering Parameters.

Sintering Parameters	Original Sintering	Extended Sintering
Heating Time to Sintering Temperature (T_HT_)	100 min	120 min
Sintering Time at Temperature (t_S_)	90 min	150 min
Sintering Temperature (T_S_)	450 °C	480 °C
Cooling Rate to Room Temperature (t_CT_)	90 min	110 min
Reheat Rate to Testing Temperature (t_RHT_)	60 min	60 min
Cooling Rate to Room Temperature to Measure Dimensions (t_CR_)	30 min	30 min

## Data Availability

Data are contained within the article.

## Appendix A

**Table A1:** Mechanical Testing and Sintering Data.

Sample ID	Ash Type	Mechanical Testing Parameters					Sintering Parameters		Geometric Parameters				
		Testing Temperature, T_T_ (°C)	Maximum Breaking Load, *F_max_* (N)	$\mathrm{Maximum}\;\mathrm{Extension},\;\delta_{max}\;(\mathrm{mm})$	Critical Stress Intensity Factor, K_c_ (MPa·mm^1/2^)	Fracture Behaviour	Sintering Time, t_S_ (h)	Sintering Temperature, T_S_ (°C)	Post-Sintering Specimen Thickness, t_S_ (cm)	Post-Sintered Notch Length, a_S_ (cm)	$\mathrm{Pre-Sintered}\;\mathrm{Density},\;\rho_{o}\;(g/{\mathrm{cm}}^{3})$	$\mathrm{Post-Sintered}\;\mathrm{Density},\;\rho_{S}\;(g/{\mathrm{cm}}^{3})$	$\mathrm{Degree}\;\mathrm{of}\;\mathrm{Sin}\mathrm{tering},\;\rho_{S}/\rho_{o}$
**1.1**	Mill A	22	50.5	0.630	6.31	Brittle	1.5	450	1.00	0.300	0.890	1.81	2.04
**2.1**	Mill A	500	2.91	6.52	0.418	Ductile	1.5	450	1.05	0.300	0.895	2.31	2.56
**2.2**	Mill A	400	37.3	0.435	6.59	Brittle	1.5	450	0.861	0.325	0.897	1.86	2.08
**3.1**	Mill A	400	51.3	0.680	6.60	Brittle	1.5	450	1.05	0.305	0.921	2.02	2.19
**3.3**	Mill A	400	16.3	0.555	6.63	Brittle	1.5	450	1.08	0.870	0.904	1.80	1.99
**4.1**	Mill A	475	11.9	6.43	2.16	Ductile	1.5	450	0.824	0.350	0.981	2.12	2.16
**4.2**	Mill A	475	14.8	6.73	2.48	Ductile	1.5	450	0.794	0.311	0.893	1.87	2.09
**4.3**	Mill A	475	18.7	6.57	3.09	Ductile	1.5	450	0.784	0.307	0.961	2.02	2.10
**5.1**	Mill A	460	21.1	6.68	3.92	Ductile	1.5	450	0.800	0.354	0.928	2.04	2.20
**5.2**	Mill A	460	36.8	7.03	4.95	Ductile	1.5	450	1.01	0.304	0.921	2.06	2.23
**5.3**	Mill A	460	41.8	6.91	4.88	Ductile	1.5	450	1.15	0.307	0.919	2.00	2.18
**6.1**	Mill B	400	60.6	0.670	6.84	Brittle	1.5	450	1.19	0.300	0.810	1.72	2.12
**6.2**	Mill B	460	28.6	7.01	4.19	Ductile	1.5	450	1.01	0.329	0.949	2.17	2.28
**6.3**	Mill B	475	27.4	7.02	3.36	Ductile	1.5	450	1.17	0.301	0.916	2.01	2.19
**6.4**	Mill B	500	4.08	6.95	0.603	Ductile	1.5	450	0.980	0.310	0.875	2.15	2.45
**7.1**	Mill B	400	63.3	0.410	8.01	Brittle	2.5	480	1.18	0.312	0.908	1.89	2.10
**7.2**	Mill B	460	50.8	0.640	7.37	Brittle	2.5	480	1.05	0.321	0.912	2.19	2.40
**7.3**	Mill B	475	34.7	0.850	4.93	Brittle	2.5	480	1.14	0.326	0.926	2.27	2.46
**7.4**	Mill B	500	5.45	1.84	0.834	Ductile	2.5	480	0.890	0.265	0.897	2.15	2.40
**8.1**	Mill A	440	29.8	0.605	5.51	Brittle	1.5	450	0.767	0.305	0.906	1.81	2.00
**8.2**	Mill A	440	44.4	1.01	6.01	Brittle	1.5	450	1.10	0.315	0.845	1.66	1.96
**8.3**	Mill A	450	32.1	6.96	5.31	Ductile	1.5	450	0.887	0.345	0.893	1.79	2.01
**9.1**	Mill A	400	63.9	2.11	7.09	Brittle-Slow	1.5	450	1.15	0.301	0.806	1.52	1.91
**9.2**	Mill A	450	17.2	0.585	4.12	Brittle	1.5	450	0.601	0.328	0.878	2.15	2.15
**9.3**	Mill A	450	29.8	2.32	4.25	Ductile-Brittle	1.5	450	0.762	0.326	0.884	1.89	2.14
**9.4**	Mill A	475	36.5	0.745	4.81	Brittle-Fast	1.5	450	0.984	0.302	0.801	1.72	2.15
**10.1**	Mill A	400	40.3	0.660	6.59	Brittle-Fast	1.5	450	0.901	0.350	0.949	2.32	2.44
**10.2**	Mill A	450	22.8	0.360	4.37	Brittle-Fast	1.5	450	0.703	0.329	0.873	2.20	2.52
**10.3**	Mill A	490	27.4	0.465	3.33	Brittle-Fast	1.5	450	1.07	0.303	0.936	2.11	2.26
**10.4**	Mill A	500	19.3	0.460	2.19	Brittle-Fast	1.5	450	1.26	0.321	0.923	1.98	2.15
